# Sustainability of rural tourism in poverty reduction: Evidence from panel data of 15 underdeveloped counties in Anhui Province, China

**DOI:** 10.1371/journal.pone.0283048

**Published:** 2023-03-13

**Authors:** Fuwei Wang, Lei Du, Minghua Tian, Yi Liu, Yichi Zhang

**Affiliations:** 1 School of Economics and Management, Beijing Forestry University, Beijing, P.R. China; 2 China Construction Engineering Design and Research CO., LTD, Beijing, P.R. China; 3 Service Center for Overseas Students and Experts, The Ministry of Human Resources and Social Security of the People’s Republic of China, Beijing, P.R. China; CRIDA: Central Research Institute for Dryland Agriculture, INDIA

## Abstract

Based on the characteristics of underdeveloped areas, this paper selects the panel data of 15 underdeveloped counties in Anhui Province from 2013 to 2019 and uses the panel threshold model to empirically analyze the sustainability of rural tourism development. The results show that: (1) Rural tourism development has a non-linear positive impact on poverty alleviation in underdeveloped areas and has a double threshold effect. (2) When the poverty rate is used to express the poverty level, it can be found that the development of rural tourism at a high level can significantly promote poverty alleviation. (3) When the number of poor people is used to express the poverty level, it can be found that the poverty reduction effect shows a marginal decreasing trend with the phased improvement of the development level of rural tourism. (4) The degree of government intervention, industrial structure, economic development, and fixed asset investment play a more significant role in poverty alleviation. Therefore, we believe that we need to actively promote rural tourism in underdeveloped areas, establish a mechanism for the distribution and sharing of rural tourism benefits, and form a long-term mechanism for rural tourism poverty reduction.

## 1 Introduction

Tourism is not only an important economic sector for many developing countries and a potential driving force for economic development but also plays an increasingly important role in poverty eradication and improving people’s livelihood [1, 2]. According to the data released by the World Travel & Tourism Council (WTTC) in 2021, before the outbreak of COVID-19, the contribution of tourism to global GDP was 10.4% (the US $9.2 trillion), accounting for 10.6% (334 million) of all jobs. The countryside is a regional complex with natural, social, and economic characteristics. It has multiple functions such as production, life, ecology, and culture and constitutes an advantageous condition for poverty alleviation through tourism. [3]. As the largest developing country in the world, China has made many policy explorations and path innovations in developing rural tourism poverty alleviation and has achieved good poverty reduction results [4]. *The 14th five-year plan for national economic and social development of the people’s Republic of China* and *The outline of long-term goals for 2035* point out that it is necessary to expand leisure agriculture, rural tourism, and other characteristic industries, consolidate and enhance the achievements of poverty alleviation, improve the overall development level of poverty alleviation areas, and realize the effective connection between consolidating and expanding the achievements of poverty alleviation and rural revitalization. Rural tourism is a meaningful way to maintain the achievements of poverty eradication, reduce relative poverty and realize rural revitalization. Therefore, studying the poverty reduction effect of rural tourism development is of great significance for giving full play to the role of rural tourism in maintaining the achievements of poverty eradication, reducing relative poverty, and realizing rural revitalization.

The research on tourism poverty reduction began with Peters [5] and Kadt and Jehuda [6]. They mainly studied the role, significance, and negative impact of tourism poverty reduction on the social and economic development of the host country. However, most of the literature on the impact of poverty on the tourism multiplier effect and economy does not take poverty as the research center, nor do they take poverty and promoting the development of poor people as the research objectives [7]. DFID proposed the concept of PPT (pro-poor tourism) in the Commission on sustainable development report, "tourism for the poor," which directly connects tourism development with poverty eradication. The international community has widely supported the concept, and since then, the academic community has begun to conduct in-depth research.

The understanding of the poverty reduction effect of tourism has formed three distinct viewpoints: (1) Tourism development alleviates poverty [8, 9] and is an effective tool for poverty alleviation in developing countries [10, 11]. (2) Tourism development has aggravated poverty [12, 13] and widened the gap between the rich and the poor [13–17]. (3) Tourism development is not necessarily related to poverty alleviation [18], and there is no convincing evidence [19, 20]. In recent years, against the background of poverty alleviation in China, the poverty reduction effect of tourism has also become one of the hot spots in domestic research, which also provides new ideas and new evidence for studying the poverty reduction effect of tourism.

Based on the existing research, the scholars’ views on the poverty reduction effect of tourism development and the research on its occurrence mechanism can be summarized into three aspects: (1) Most scholars believe that tourism can directly alleviate poverty by lowering the employment threshold [21, 22] and providing jobs [23, 24], driving the development of relevant industries [25–28], increasing the income of the poor [2, 29–31], and improving transportation conditions [32]. Tourism development also has a "multiplier effect" on economic growth [9, 33, 34], thus indirectly alleviating poverty through the "trickle-down effect" [33]. (2) Some scholars believe that tourism development in areas with a small scale is prone to the phenomenon of "tourism leakage," in which tourism income flows to foreign countries [35, 36]. This phenomenon has aggravated poverty [12, 20] and led to a widening gap between the rich and the poor [13–17]. At the same time, tourism development is affected by many factors, such as the control of foreign capital [16, 36], the lack of control over resources [14], and the level of infrastructure construction [37]. Suppose the income distribution mechanism is not perfect. In that case, it will lead to the "Matthew effect", which goes against the original intention of the poor groups to eliminate poverty as a whole [20, 37, 38]. In addition, the blind pursuit of development quantity and economic benefits will also cause environmental pollution and ecological damage [39], quickly lead to poverty, and even hinder the development of agriculture and other industries [40, 41]. (3) The relationship between tourism development and poverty alleviation is complex and is not a simple "positive or negative" relationship. Underdeveloped areas may embark on the road of becoming rich through tourism development and may also enter the "development trap" [40]. Rural tourism poverty alleviation has three effects: economic, social, and environmental. The three effects have both positive and negative effects [42]. Moreover, there are conflicts between poverty reduction by tourism and poverty caused by tourism in the dimensions of economy, culture and psychology, social relations, and rights [43]. The poverty reduction effect of tourism is affected by the diversity of local tourism resources, initial facilities, service level, consumption capacity, and other factors, showing apparent spatial heterogeneity [19, 44]. The relationship between them is nonlinear and has a significant threshold effect.

It can be seen that scholars have different views on the poverty reduction effect of tourism development. Even though it is believed that the poverty reduction effect of tourism has heterogeneity, non-linear characteristics, or threshold effect, most of them are based on provincial data and lack specific guidance for specific regions. In addition, most of the studies focus on the tourism development of the whole region rather than rural tourism, which is divorced from the reality of poverty reduction and poverty alleviation in rural areas. Moreover, the poverty level of some studies is estimated or used as a proxy indicator, which may be out of line with the actual situation and affect the reliability of the results. To sum up, there are few studies on the poverty reduction effect of tourism based on the specific development of specific regions in the existing literature, and the research based on the underdeveloped counties of a specific province and from the perspective of rural tourism rather than tourism is even less. Is rural tourism sustainably for poverty alleviation in underdeveloped areas? How effective is it? Is there a threshold? What is the threshold? What is the impact of different threshold intervals? Does it have a marginal diminishing effect? Both need to be studied in depth.

The marginal contribution of this paper is mainly reflected in the following four aspects: (1) This paper studies the sustainability of rural tourism to poverty reduction in underdeveloped areas and expands and refines the research field of tourism poverty reduction effect. (2) Based on a systematic analysis of the literature on the poverty reduction effect of tourism, we propose that the poverty reduction effect of rural tourism is sustainable and has a threshold effect. (3) When examining the poverty reduction effect of rural tourism, we should use the poverty incidence rate and the number of poor people in the country to measure the poverty level rather than estimate or use substitute indicators. Therefore, we propose to express the development level of rural tourism by the total income of rural catering and accommodation /GDP rather than the total income of regional tourism /GDP. (4) When constructing the threshold model, we propose a control index system for poverty alleviation in underdeveloped areas. This aligns with the reality of underdeveloped areas with the county as the research unit.

The rest of this paper is arranged as follows: Section 2 is the theoretical analysis and research hypothesis; Section 3 is the research methods and data; Section 4 is the empirical results and discussion; Section 5 is the conclusion and policy recommendations.

## 2 Theoretical analysis and research hypothesis

The theory of poverty reduction caused by the positive effect of tourism poverty reduction and the theory of poverty caused by the negative effect is undoubtedly one-sided. The positive and negative effects of tourism poverty reduction always exist and are constantly changing, so the poverty reduction effect of rural tourism is complex.

Some scholars believe that the "multiplier effect" and "leakage effect" always accompany each other [45]. Therefore, one-sided emphasis on one aspect or solidification of its role is undoubtedly undesirable. As mentioned above, the tourism poverty reduction effect has spatial heterogeneity, time nonlinearity, and threshold effect, which some studies have confirmed. Its root cause is the complexity of tourism poverty reduction effect. However, the common problem in the existing research on the threshold effect of tourism poverty reduction is that the research area is too macro, and there is no research on rural tourism. The selection of indicators remains to be discussed or does not apply to small regions below the provincial level, especially rural and underdeveloped areas. Therefore, to consolidate the requirements of poverty alleviation, it is necessary to take rural tourism, closely related to rural poverty reduction, as the research object for specific underdeveloped areas with a high risk of returning to poverty. Based on its actual level of rural tourism development, considering other regional specific factors that affect the level of poverty, research the comprehensive effect of rural tourism development on poverty alleviation to have targeted and specific guiding significance.

**Fig 1:**
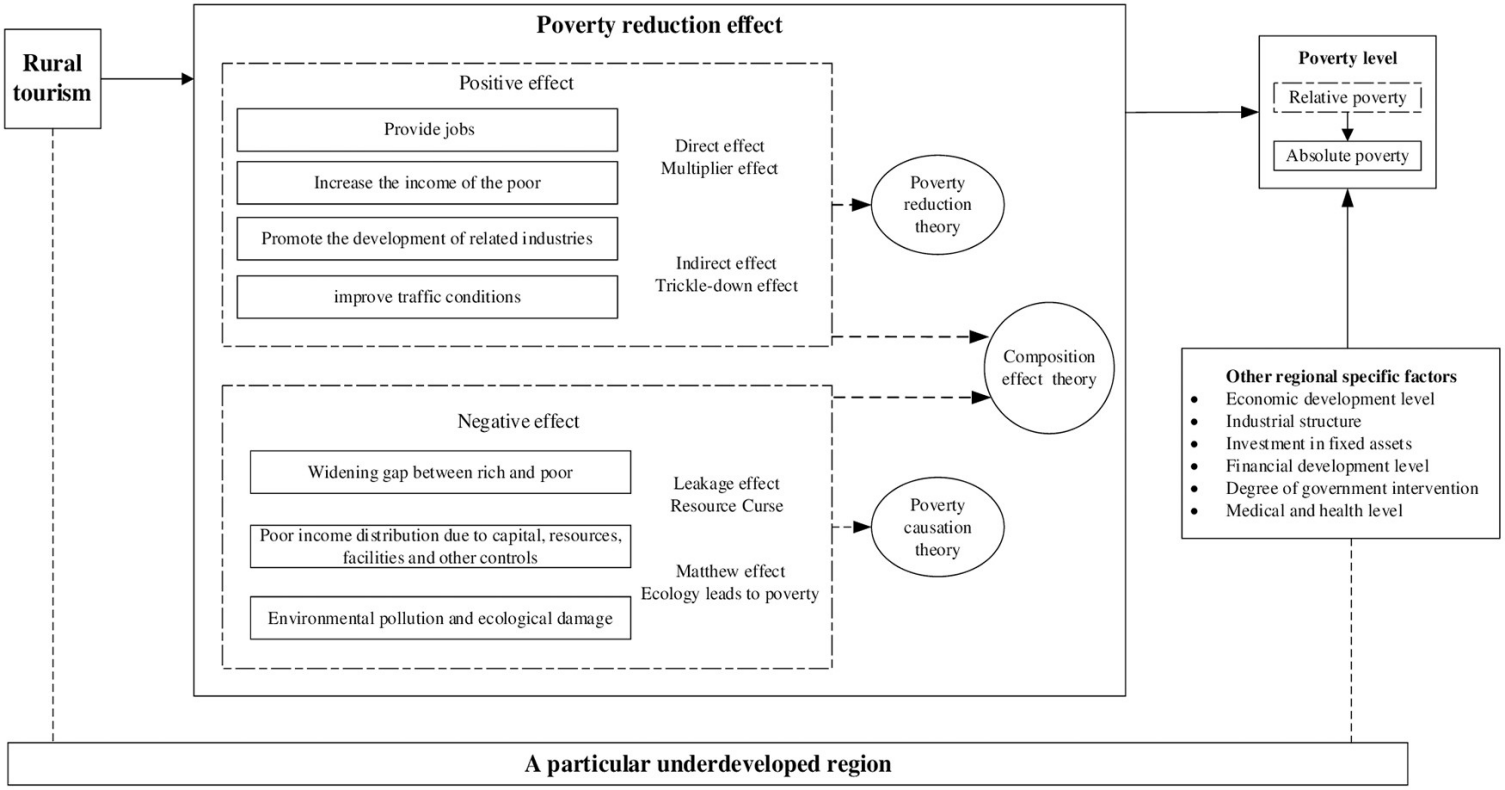
Theoretical analysis framework.

The theoretical framework of this paper is shown in Fig 1. According to the theory of tourism destination life cycle, the development level of rural tourism in underdeveloped areas will be gradually improved with the tourism development, especially the local tourism development of foreign capital. Therefore, the "multiplier effect" of rural tourism is becoming more and more significant, especially with the development of rural tourism. The income distribution mechanism of rural tourism development continues to improve, weakening the "leakage effect" and "Matthew effect" of rural tourism. Therefore, the poverty reduction effect of rural tourism is significantly different due to different stages of rural tourism development. Accordingly, this paper puts forward the research hypothesis H1.

**H1**: **Rural tourism is sustainable for poverty reduction in undeveloped areas and has a threshold effect**.

The hypothesis that tourism development promotes economic growth has been recognized by scholars at the theoretical and empirical levels [33]. Therefore, it is reasonable to believe that the development of rural tourism will also have a positive impact on the economic growth of underdeveloped areas and then benefit the vast number of poor people through the "trickle-down effect" of economic growth. However, rural tourism development does not always cause sustained and accelerated economic growth, so the indirect "trickle-down effect" is weakening. From the perspective of the direct effect of rural tourism on poverty reduction, with the improvement of the development level of rural tourism, rural tourism has been transformed and upgraded from extensive operation to intensive scale, the threshold of employment has been raised, and the relative number of people who absorb employment has decreased. Therefore, the poverty reduction effect of rural tourism may present a marginal decline trend. Accordingly, this paper proposes the research hypothesis H2.

**H2**: **The poverty reduction effect of rural tourism decreases marginally with the improvement of rural tourism development level**.

## 3 Methodology and data

### 3.1 Model established

Due to the poverty reduction effect of rural tourism may differ in different stages of rural tourism development, the commonly used multiple linear regression method should not be used. In addition, because it is impossible to determine the stage dividing point of rural tourism development in advance, it is also difficult to adopt the breakpoint regression analysis method. Therefore, this paper conducts empirical research based on the panel threshold model proposed by Hansen [46], Hansen [47], Hansen [48]. The threshold effect is the different influence of the value of threshold variables in different intervals on the explained variables. Therefore, in this paper, the panel threshold effect model of Hansen [47] is used to analyze, and the bootstrap method is used to evaluate the statistical significance of the threshold effect [49] to determine whether there is a threshold effect. If there is a threshold effect, determine the threshold value and then conduct regression according to the threshold partition to find the impact of the threshold variable on the explained variable in different intervals. The specific formula is:

$$y_{it}=\mu_{i}+\beta_{1}ts_{it}I\left(ts_{it}\leq \gamma_{1}\right)+\cdots +\beta_{n+1}ts_{it}I\left(ts_{it}>\gamma_{n}\right)+\delta X_{it}+\varepsilon_{it}$$ (1)

In formula (1): *i* represents the observed individual; *t* represents the observation time; *y*_it_ refers to the poverty level; *ts*_it_ refers to the development level of rural tourism; *γ*_1_, …, *γ*_n_ is the threshold value, and n threshold values can divide the threshold variable into n+1 threshold regions; *I*(*·*) is an indicative function, if the threshold variable meets the conditions, it is taken as 1, otherwise it is 0; *X*_it_ is the set of control variables; *μ*_*i*_ represents the non-observed fixed effect that does not change with time; *ε*_*it*_ is a random disturbance term and follows the standard normal distribution.

### 3.2 Variable selection

#### 3.2.1 Explained variable

Poverty level (y) is the explanatory variable of this paper. Scholars usually use poverty incidence [50], poverty income level [4, 38], the Engel coefficient [51, 52], the poverty FGT index [53], and other indicators to measure poverty level. Since the research area of this paper is 15 underdeveloped counties in Anhui Province, and the poverty population has been accounted for according to the unified national filing and card establishment. Therefore, we believe that the poverty incidence index is more accurate. In addition, the number of poor people (10000 people) in 15 underdeveloped counties in Anhui Province is set as a proxy index to measure the degree of poverty for the robustness test.

#### 3.2.2 Core explanatory variable and threshold variable

Rural tourism development level (ts) is the core explanatory variable and threshold variable of this paper. Previous studies have used tourism specialization indicators to measure the level of rural tourism development [54, 55], and the calculation formula is tourism specialization degree = total tourism revenue /GDP. However, the total tourism revenue, including urban and even international tourism, cannot represent the development level of rural tourism. In addition, due to the lack of total county tourism revenue data of the less developed counties in the study area, the survey found that the rural tourism revenue of the less developed counties in Anhui Province mainly comes from catering and accommodation revenue. This data is available. Therefore, we use the total rural catering and accommodation revenue data to replace it. That is, ts = total income of rural catering and accommodation /GDP. When rural tourism development is at different levels, the impact on the employment and income of the poor is different, resulting in a threshold effect.

#### 3.2.3 Control variables

In order to avoid endogenous problems caused by missing variables, we also set the relevant factors affecting the development of rural tourism and poverty alleviation as control variables. The details are as follows:

1. Economic development level (PGDP). We use the per capita GDP of the less developed counties in Anhui Province (10000 yuan) to express this variable. Economic growth can improve the average income level, which will positively affect the income of the poor, thus reducing poverty. Therefore, in the study of the tourism poverty reduction effect, the level of economic development is the primary control variable. Generally speaking, the higher the level of economic development, the lower the poverty level.
2. Industrial structure (FIS). We use the output value of the primary industry / GDP of each county to express this variable. We use the proportion of the added value of the primary industry as the proxy variable of industrial structure. This is because poverty mainly occurs in rural areas, and the poor cannot participate in the secondary and tertiary industries for various reasons. On the contrary, they rely more on the primary industry.
3. Fixed asset investment (PFA). We use the fixed asset investment of each county / regional GDP to express this variable. Most of the literature on poverty alleviation takes the level of fixed asset investment as a control variable and usually shows the significance of Poverty Alleviation, so we introduce it as a control variable.
4. Financial development level (FS). We use the annual loan balance / annual GDP of each county to express this variable. There are many studies on financial poverty alleviation and poverty reduction. Although the empirical test conclusions are different, it is enough to explain that financial development is an essential factor affecting poverty alleviation.
5. Degree of government intervention (PG). We use the fiscal expenditure / GDP of each county to express this variable. Poverty alleviation is an essential work of the government and even becomes the primary task in the poverty eradication stage, but the government’s financial resources limit it. Generally speaking, the greater the government’s financial resources, the stronger the poverty alleviation and reduction.
6. Health care level (MH). To express this variable, we use the number of beds in medical and health institutions in each county (10000). Many poor people are poor because of illness and then get poorer because of poverty. The level of medical and health care should have a particular impact on the occurrence and reduction of poverty.

### 3.3 Study area, data sources and statistical description

We take the county panel data of 15 underdeveloped counties in Anhui Province in the poverty alleviation stage as samples, including Dangshan, Huoqiu, Jinzhai, Lixin, Lingbi, Qianshan, Shitai, Shouxian, Shucheng, Sixian, Taihu, Wangjiang, Xiaoxian, Susong and Yuexi. The reasons for choosing Anhui Province as the research sample are as follows:

Anhui is a province with a heavy task of poverty alleviation, involving 16 provincial-level cities and 70 counties (cities and districts), including 20 national-level poverty counties and 11 provincial-level poverty counties. In 2014, 3000 poverty-stricken villages and 4.84 million poverty-stricken people were identified by filing cards. In 2015, the National Tourism Administration issued the Notice on the Establishment of the "National Territorial Tourism Demonstration Zone", and all parts of Anhui Province began to actively carry out the establishment of the National Territorial Tourism Demonstration Zone. In June 2016, Anhui Province issued the Administrative Measures for Tourism Standardization (Trial), focusing on the construction of tourism standardization, speeding up the pace of tourism transformation and development, and vigorously promoting regional tourism. In 2016, the National Tourism Administration announced the list of national tourism demonstration zones. There were 262 in the first batch, 2 prefecture-level cities and 9 county-level cities and counties in Anhui Province, 238 in the second batch, and 1 prefecture-level city and 10 county-level cities and counties in Anhui Province. In view of the availability of data, it is difficult to obtain complete data of all 31 poor counties in Anhui Province. Therefore, this paper selected the county panel data of 15 underdeveloped counties in Anhui Province in the poverty alleviation stage as samples.

Since rural tourism was seriously affected by COVID-19, the data range is 2013–2019. The original data comes from the government work reports of all counties over the years, the Statistical Bulletins of national economic and social development, and the statistical yearbooks and survey data at the provincial and municipal levels. In order to reduce the influence of dimension and heteroscedasticity and facilitate the elastic analysis in the economic sense, we take logarithms of all variables. The descriptive statistical analysis results of each variable are shown in Table 1.

**Table 1:** Descriptive statistical analysis of variables.

Variables	Symbol	Obs	Mean	Std.Dev.	Min	Max
Poverty level	*y*	105	0.0790	0.0689	0.0000	0.3010
Rural tourism development level	*ts*	105	0.0233	0.0092	0.0087	0.0509
Economic development level	*PGDP*	105	1.7947	0.5548	0.8328	3.5268
Industrial structure	*FIS*	105	0.2225	0.0574	0.1109	0.3521
Investment in fixed assets	*PFA*	105	0.9233	0.3421	0.3529	2.2780
Financial development level	*FS*	105	0.7552	0.2832	0.2977	1.8325
Degree of government intervention	*PG*	105	0.2835	0.0832	0.1901	0.5772
Medical and health level	*MH*	105	0.2350	0.1098	0.0323	0.4780

## 4 Empirical results and discussions

### 4.1 Threshold effect existence test

In order to avoid the phenomenon of pseudo regression, this paper first uses the Hadri test to test the stability of panel data. The results are shown in Table 2. It can be seen from Table 2 that all variables are stable at the significance level of 1%.

**Table 2:** Unit root test.

Variables	Hadri LM test	P-value	Result
ln*y*	11.3805***	0.0000	Stable
ln*ts*	4.8131***	0.0000	Stable
ln*PGDP*	9.7622***	0.0000	Stable
ln*FIS*	10.6171***	0.0000	Stable
ln*PFA*	6.1430***	0.0000	Stable
ln*FS*	9.8855***	0.0000	Stable
ln*PG*	4.0339***	0.0000	Stable
ln*MH*	8.5204***	0.0000	Stable

Notes:

*, **, *** represent the significance at 10%, 5%, and 1%, respectively.

Taking the rural tourism development level as the threshold variable, we use the bootstrap method to test the existence of the threshold effect. The results are shown in Table 3. From Table 3, we can find that both single and double threshold tests are significant. The single threshold is significant at 10%, and the double threshold is significant at 1%. Therefore, the double threshold is more significant than the single threshold. We believe there is a double threshold effect.

**Table 3:** Sample inspection results of threshold effect.

Number of thresholds	F-Value	P-Value	BS times	Critical value		
				1%	5%	10%
Single threshold	18.0800*	0.0625	400	28.6338	19.4940	14.1820
Double threshold	26.2300***	0.0050	400	21.2206	17.3819	13.2069
Triple threshold	20.1500	0.4375	400	77.9144	55.4885	42.5477

Note: P value is the result of repeated sampling 400 times by bootstrap method;

*, **, *** represent the significance at 10%, 5%, and 1%, respectively.

Assuming that there is a double threshold effect, we estimate that the two thresholds are 0.0244 and 0.0374, respectively, and the corresponding 95% confidence intervals are [0.0231,0.0253] and [0.0360,0.0376], respectively. This proves that the research hypothesis H1 is true. That is, the development of rural tourism has a threshold effect on poverty reduction in underdeveloped areas. The data shows that the development level of rural tourism in 15 underdeveloped counties in Anhui Province is relatively low. According to the data of Anhui Province, under the same caliber, the proportion of rural tourism income in GDP in underdeveloped counties is generally lower than that in non-underdeveloped counties, and there is a big gap with the national level. Even so, we found that the development level of rural tourism in underdeveloped counties in Anhui Province still has phased characteristics. According to two thresholds, the development level of rural tourism in underdeveloped counties in Anhui Province can be divided into three stages: in the first stage, ln*ts* ≤ 0.0244, rural tourism development is relatively low; In the second stage, 0.0244< ln*ts* ≤ 0.0374, the development of rural tourism is relatively at a medium level; In the third stage, ln*ts* >0.0374, the development of rural tourism is at a relatively high level.

### 4.2 Panel threshold effect regression results

Based on determining the existence of double thresholds, we divide the two thresholds obtained into three intervals for panel threshold effect regression. The results are shown in Table 4.

**Table 4:** Regression estimation results of panel threshold model.

Variables	Coefficient	P-value
*μ*_*i*_	0.4500***	0.0000
ln*ts*≤0.0244	0.0159	0.9790
0.0244<ln*ts*≤0.0374	2.5769	0.1590
ln*ts*>0.0374	-0.6725**	0.0430
ln*PGDP*	-0.2643***	0.0000
ln*FIS*	-0.3748**	0.0150
ln*PFA*	-0.0984***	0.0050
ln*FS*	-0.0619	0.1220
ln*PG*	-0.5174***	0.0010
ln*MH*	0.2013	0.2010

Notes:

*, **, *** represent the significance at 10%, 5%, and 1%, respectively.

In the first and second stages, the development of rural tourism has no significant impact on poverty alleviation and even exacerbated poverty in terms of impact coefficient. With the development level of rural tourism rising to the third stage, it has a significant role in promoting poverty alleviation. The development of rural tourism increased by 1%, and the poverty rate decreased by 0.6725%. The results show differences in the poverty reduction role of rural tourism development in underdeveloped areas at different stages, which further confirms the validity of the research hypothesis H1.

On the one hand, when the level of rural tourism development is low, the excellent tourism resources in underdeveloped areas have not been effectively developed, and the resource advantages cannot be transformed into economic benefits. In addition, the overexploitation of resources destroys the ecological environment, resulting in poor poverty reduction and even the phenomenon of poverty alleviation. When the types of tourism resources are consistent, people are more inclined to choose tourist destinations with short time-consuming, and high accessibility. Most of the underdeveloped areas are located on the periphery of the city. It is challenging to complete the improvement of transportation facilities in the initial stage, thus weakening the advantages of tourism resources in poor areas. On the other hand, from the perspective of micro individuals, in the early stage of rural tourism development, the first people who can participate by opening restaurants and hotels are often non-disabled families with capital in the village. Due to the lack of funds and skills, the benefits of the poor are often excluded and unevenly distributed. In addition, foreign investors with capital and market experience advantages will also seize the benefit opportunities of the poor. In the mature stage of rural tourism development, more and more poor people participate in rural tourism and escape poverty. Foreign capital enters and develops rural tourism. Due to policy intervention, local tourism enterprises have provided many jobs for the poor. In addition, transportation facilities have been gradually improved to encourage more urban people to go deep into rural tourism. These reasons make rural tourism play a significant role in poverty alleviation when it reaches a high level.

As for the control variables, government intervention is essential in promoting poverty alleviation. For every 1% increase in government intervention, the poverty rate will drop by 0.5174%. This is in line with the reality of development. On the one hand, the higher the degree of government intervention, the more financial support for the poor can directly alleviate poverty. On the other hand, government intervention will also indirectly guide social capital to flow to the field of poverty alleviation, improve the efficiency of fund allocation, and better serve poverty alleviation. The second is the industrial structure. For every 1% increase in the industrial structure, the poverty rate will drop by 0.3748%. This is because the cost of the population in the underdeveloped areas participating in the economic activities of the secondary and tertiary industries is high, and the poor people are mainly staying in the primary industry. Therefore, the development of the primary industry is more conducive to poverty alleviation. However, due to the prominent contradiction between people and land, the difficulty in realizing large-scale operations, the insufficient accumulation of human capital, and other practical factors, the development of the primary industry is difficult to break through the bottleneck. To enhance the poverty reduction effect of the primary industry, it is necessary to fully tap the local natural ecological, historical and cultural tourism resources to develop rural tourism. In this way, people can realize development transformation and increase income without leaving their places of residence. At the same time, in developing tourism resources, it is still necessary to protect the development of the primary industry and must not be blind and extreme. Then there is the level of economic development. Every 1% increase in the level of economic development will reduce the poverty rate by 0.2643%. This is mainly due to the improvement of economic development, which has continuously improved the infrastructure construction of transportation, post and telecommunications, power supply, and water supply, thus providing convenience for developing rural tourism and other industries. In this way, the market is more dynamic, providing more employment opportunities and higher labor remuneration, thus promoting poverty alleviation. Finally, for every 1% increase in fixed asset investment, the poverty rate will drop by 0.0984%. The investment environment in the underdeveloped areas is poor, the investment level is low, and the investment projects are mainly concentrated in the regional centers. Therefore, it plays a relatively minor role in alleviating regional poverty, especially rural poverty.

The level of financial development and medical care has little impact on poverty alleviation. This is mainly due to the low level of financial development in the underdeveloped areas, and the poor people cannot afford the costs related to the services of financial institutions. Even if they participate in developing the financial industry, they will be disadvantaged in the distribution of interests. The unbalanced distribution of interests and financial fluctuations will significantly offset the poverty alleviation effect brought by financial development. The medical and health level is not significant, indicating that the improvement of medical and health conditions has not benefited the poor. It should be because poor people cannot pay for medical services because of poverty.

### 4.3 Endogenous problems

The current value of rural tourism development level (ln*ts*) and model interference(*ε*). The current value may be related, thus generating endogenous problems. In order to reduce the impact of endogenous problems, we replace the current value of ln*ts* with the lag value of ln*ts* for the existence test of the threshold effect and the regression analysis of the panel threshold model. The results are shown in Tables 5 and 6. It can be seen that after the development level of rural tourism lags behind for a period, it will still have a nonlinear impact on the poverty level with double thresholds. The estimated results of the two thresholds and the inter-regional threshold effect are consistent with the current value, indicating that the endogenous problem of the rural tourism development level variable has a small impact.

**Table 5:** Results of the self-sampling test on threshold effect of core explanatory variables lagging behind one period.

Number of thresholds	F-Value	P-Value	BS times	Critical value		
				1%	5%	10%
Single threshold	10.6700	0.2275	400	38.1631	20.5933	16.3247
Double threshold	25.1400***	0.0025	400	20.5658	16.5215	13.9347
Triple threshold	6.9200	0.3875	400	25.4401	18.2599	13.6226

Note: P value is the result of repeated sampling 400 times by bootstrap method;

*, **, *** represent the significance at 10%, 5%, and 1%, respectively.

**Table 6:** Estimation results of panel threshold model parameters with core explanatory variables lagging behind one period.

Variables	Coefficient	P-value
Number of thresholds	2***	0.0030
threshold	0.0244 and 0.0374	
*μ*_*i*_	0.4064***	0.0000
ln*ts*_*it*_≤0.0244	0.2076	0.7770
0.0244<ln*ts*_*it*_≤0.0374	1.5693	0.2640
ln*ts*_*it*_>0.0374	-1.0641**	0.0380
ln*PGDP*_*it*_	-0.2219***	0.0000
ln*FIS*_*it*_	-0.5622***	0.0020
ln*PFA*_*it*_	-0.1389***	0.0010
ln*FS*_*it*_	-0.0413	0.3430
ln*PG*_*it*_	-0.5630***	0.0010
ln*MH*_*it*_	0.1813	0.2590
Effective sample size	90	

Notes:

*, **, *** represent the significance at 10%, 5%, and 1%, respectively.

### 4.4 Robustness test

#### (1) Remove one control variable from the panel threshold model

The economic development level (ln*PGDP*), industrial structure (ln*FIS*), fixed asset investment level (ln*PFA*), financial development level (ln*FS*), government intervention level (ln*PG*), and medical and health level (ln*MH*) are removed from the panel threshold model one by one for regression analysis. The results are shown in Table 7.

**Table 7:** Regression results with one control variable removed one by one.

	Remove	Remove	Remove	Remove	Remove	Remove
	ln*PGDP*	ln*FIS*	ln*PFA*	ln*FS*	ln*PG*	ln*MH*
Number of threshold values	2	2	2	2	2	2
Threshold	0.0244	0.0244	0.0244	0.0244	0.0244	0.0244
	0.0374	0.0374	0.0374	0.0374	0.0374	0.0374
*μ*_*i*_	0.5601***	0.5805***	0.4596***	0.4793***	0.4021***	0.4551***
ln*ts* in the first section	0.2761	0.1494	0.0418	0.0913	0.2023	0.1706
ln*ts* in the second section	2.7261	2.8483	2.6151*	1.7609	2.9909	2.6139
ln*ts*in the third section	-0.9276*	-0.4632***	-0.8140*	-0.8394*	-0.5165*	-0.7542**
ln*PGDP*		-0.3282***	-0.2782***	-0.2919***	-0.2588***	-0.2437***
ln*FIS*	-0.1860***		-0.0329*	-0.0442***	-0.2410	-0.3402**
ln*PFA*	-0.1326***	-0.0897		-0.1058***	-0.1410***	-0.0917**
ln*FS*	-0.0548	-0.0964*	-0.0724*		-0.1302**	-0.0290
ln*PG*	-0.4686**	-0.4241***	-0.6702***	-0.6888***		-0.5260***
ln*MH*	0.0434	0.1805	0.1804	0.2041	0.2088	

Notes:

*, **, *** represent the significance at 10%, 5%, and 1%, respectively.

Table 7 shows that there is still a double threshold for the role of rural tourism development level on the poverty level, and the threshold value has not changed. When the development level of rural tourism is in the third interval, it has a significant role in promoting poverty alleviation, and the impact of government intervention on the level of economic development is the same. The difference is that when ln*PFA* is removed, the level of rural tourism development in the second interval will significantly increase poverty. And when different control variables are removed, the coefficients of *lnFIS*、*lnPFA* and ln*FS* are significantly different. However, the significant coefficients all indicate that these control factors have a promoting effect on poverty alleviation.

#### (2) Replace the poverty rate with the number of poor people

In this paper, the poverty level is measured by using the number of poor people with documented cards instead of the incidence of poverty to further test the robustness of the poverty reduction effect of rural tourism. The results are shown in Tables 8 and 9.

**Table 8:** The core explanatory variable is the threshold effect of the number of poor people.

Number of thresholds	F-Value	P-Value	BS times	Critical value		
				1%	5%	10%
Single threshold	2.6760	0.1932	400	12.0314	6.5138	4.7033
Double threshold	31.9380***	0.0031	400	20.9223	11.9762	7.2688
Triple threshold	4.3830	0.2683	400	30.6979	16.0791	10.1822

**Table 9:** Regression results of poverty level expressed by poverty population.

Variables	Coefficient	P-value
*μ*_*i*_	1.6818***	0.000
ln*ts*≤0.0167	-14.2851*	0.063
0.0167<ln*ts*≤0.0234	-9.9173***	0.002
ln*ts*>0.0234	-6.1279***	0.000
ln*PGDP*	-1.0096***	0.000
ln*FIS*	-0.6488**	0.032
ln*PFA*	-0.3060*	0.083
ln*FS*	-0.1246*	0.000
ln*PG*	-0.1710***	0.007
ln*MH*	0.3449	0.409

Notes:

*, **, *** represent the significance at 10%, 5%, and 1%, respectively.

Table 8 shows that the double threshold is significant at the significance level of 1%, that is, the development level of rural tourism still shows the double threshold feature when the poverty level is measured by the number of poor people. The research hypothesis H1 is valid. It is further estimated that the two thresholds are 0.0167 and 0.0234, and the corresponding 95% confidence intervals are [0.0154,0.0182] and [0.0193,0.0291], respectively. Table 9 shows that the estimated coefficients of the development level of rural tourism in the three intervals are -14.2851, -9.9173, and -6.1279, which are significant at the significance level of 10%, 1%, and 1%, respectively. This shows that the development of rural tourism promotes reducing the number of poor people, which has a gradually decreasing trend. Therefore, the research hypothesis H2 is established. That is, the poverty reduction effect of rural tourism decreases marginally with the improvement of rural tourism development level. In Table 9, the poverty reduction effects of economic development level, industrial structure, fixed asset investment level, and government intervention level remain stable. The role of medical and health levels is still not significant. Only the financial development level has changed from insignificant to significant. Table 9 shows that from the perspective of directly reducing the number of poor people, the level of economic development and industrial structure have a greater impact. To sum up, the conclusion that rural tourism development has a double threshold effect on poverty alleviation is very stable.

## 5 Conclusion and policy recommendations

### 5.1 Conclusions

Based on the panel data of 15 underdeveloped counties in Anhui Province for seven consecutive years, this paper constructs a panel threshold regression model to empirically analyze the objective effect of rural tourism development on poverty alleviation. The results show that:

1. The development of rural tourism has a significant impact on poverty alleviation, far more than other factors. This reflects that developing rural tourism in underdeveloped areas is sustainable and effective in reducing poverty.
2. Rural tourism development in underdeveloped areas has a double threshold effect on poverty alleviation. When the poverty rate expresses the poverty level, the threshold value of the rural tourism development level is 0.0244 and 0.0374, respectively, and the rural tourism development level is in the first and second stages, which has no significant impact on poverty alleviation. As the rural tourism development level rises to the third stage, it significantly promotes poverty alleviation. When the poverty level is expressed by the number of poor people instead of the poverty rate, the threshold value of rural tourism development level is 0.0167 and 0.0234, respectively. The promotion effect of rural tourism development on reducing the number of poor people is significant in the three intervals, but the poverty reduction effect shows a marginal decreasing trend.
3. Among the control variables, the degree of government intervention, the industrial structure, the level of economic development, and the level of fixed assets investment have a more significant effect on poverty alleviation. However, the impact of financial development and medical and health levels on poverty alleviation is not apparent.

### 5.2 Policy recommendations

Based on the above conclusions, we put forward the following policy recommendations:

1. To create characteristic rural tourism and improve the development level of rural tourism to give full play to the poverty reduction effect of rural tourism. The result shows that rural tourism development plays a significant role in poverty alleviation. However, its poverty reduction role has a double threshold effect, and only at a higher level can it play a significant role in promoting. Therefore, it is the first step to rapidly improving the development level of rural tourism. This means that spontaneous rural tourism cannot develop slowly, but the government needs to promote it vigorously. According to local conditions and market demand, we will actively create distinctive rural tourism products around "food, housing, transportation, tourism, shopping, and entertainment." In order to attract more tourists to rural tourism and rapidly improve the development level of rural tourism.
2. By promoting the construction of rural tourism, the marginal decreasing trend of poverty reduction effect of rural tourism will be changed. Rural whole area tourism is to provide rural tourism products and services in all regions, all times, and all fields. Realizing the organic integration of tourism resources and the integrated development of industries can significantly increase the coverage of poverty alleviation groups, expand the regional economy, and improve the income of rural tourism. This is conducive to improving the quality of rural tourism development, slowing down, and even changing the decreasing trend of rural tourism poverty reduction. Among them, we should focus on cultivating new business forms such as agricultural tourism, leisure, health care, folk customs, and rural culture, and give play to the integrated poverty reduction effect of rural tourism.
3. Increase government intervention to optimize and upgrade the industrial structure. Strengthen infrastructure construction, improve the economic development level of underdeveloped areas, and form a synergistic effect with rural tourism to reduce poverty. The research shows that the degree of government intervention, industrial structure, economic development level, and fixed asset investment in less developed areas play a more significant role in poverty alleviation. In fact, they are not only an important way to reduce poverty, but also can promote each other with rural tourism and strengthen the poverty reduction effect of rural tourism. For example, folk cultural activities such as temple fairs and food festivals with local characteristics are held to enhance the attraction of rural tourism. Create a science and technology agricultural park integrating leisure and vacation, participation and experience to carry out "hematopoietic" industrial poverty alleviation. Develop leisure and sightseeing agriculture according to local conditions and optimize the industrial structure. Make up for the shortage of rural tourism supporting facilities such as transportation, sign guidance, communication, and e-commerce in rural areas. While playing its role in poverty reduction, It has promoted the development of rural tourism and strengthened the poverty reduction effect of rural tourism.
4. By establishing rural tourism benefit distribution and sharing mechanism, we can enhance the driving force of rural tourism development and form a long-term mechanism for rural tourism poverty reduction. We should vigorously improve the business and investment environment and encourage tourism enterprises to go to the countryside. At the same time, we should revitalize private capital, innovate rural finance, and guide capital to flow to rural tourism. While using market forces to enhance the driving force of rural tourism development, we should establish the concept of sharing, design the benefit distribution and sharing mechanism of rural tourism development, and reduce tourism leakage. Explore the mode of multi-party cooperation and joint participation, such as "base + farmers", "enterprise + farmers", "e-commerce + farmers", "village committee + cooperative + farmers + leading enterprises", "company + scientific research institute + professional cooperative + farmers", and try the benefit sharing mechanism of "guaranteed minimum dividend". Improving the dispute arbitration mechanism is also necessary to ensure that residents in less developed areas can increase their income through rural tourism.
